# HLA-DR and HLA-DP Restricted Epitopes from Human Cytomegalovirus Glycoprotein B Recognized by CD4^+^ T-Cell Clones from Chronically Infected Individuals

**DOI:** 10.1007/s10875-012-9732-x

**Published:** 2012-07-15

**Authors:** Claire Ventura, Hélène Bisceglia, Yves Girerd-Chambaz, Nicolas Burdin, Pascal Chaux

**Affiliations:** 1 Present Address: Sanofi Pasteur Biologics Co, 38 Sidney St, Cambridge, MA 02139 USA; 2 Present Address: Sanofi Pasteur, 1541 Avenue Marcel Mérieux, 69280 Marcy l’Etoile, France; 3 Discovery Department, Sanofi Pasteur, 1541 Avenue Marcel Mérieux, 69280 Marcy l’Etoile, France

**Keywords:** Cytomegalovirus, glycoprotein B, CD4^+^ T cells, HLA-class II

## Abstract

**Purpose:**

Helper CD4^+^ T cells presumably play a major role in controlling cytomegalovirus (CMV) by providing help to specific B and CD8^+^ cytotoxic T cells, as well as through cytotoxicity-mediated mechanisms. Since CMV glycoprotein B (gB) is a major candidate for a subunit vaccine against CMV, we searched for gB-epitopes presented by human leukocyte antigen (HLA)-class II molecules.

**Methods:**

Dendritic cells obtained from CMV-seropositive donors were loaded with a recombinant gB and co-cultured with autologous CD4^+^ T cells. Microcultures that specifically recognized gB were cloned by limiting dilution using autologous Epstein-Barr virus (EBV)-immortalized B cells pulsed with gB as antigen-presenting cells. To pinpoint precisely the region encoding the natural epitope recognized by a given CD4^+^ clone, we assessed the recognition of recombinant *Escherichia coli* expressing gB-overlapping polypeptides after their processing by autologous EBV-B cells.

**Results:**

We isolated several gB-specific CD4^+^ T-cell clones directed against peptides gB_190-204_, gB_396-410_, gB_22-36_ and gB_598-617_ presented by HLA-DR7, HLA-DP10 and HLA-DP2. While their precise role in controlling CMV infection remains to be established, gB-specific CD4^+^ T cells are likely to act by directly targeting infected HLA-class II cells *in vivo*, as suggested by their recognition of EBV-B cells infected by the Towne CMV strain.

**Conclusions:**

The characterization of such gB-epitopes presented by HLA-class II should help to understand the contribution of CD4^+^ T-cell responses to CMV and may be of importance both in designing a vaccine against CMV infection and in immunomonitoring of subjects immunized with recombinant gB or with vectors encoding gB.

## Introduction

Human cytomegalovirus (CMV) is a ß-herpesvirus that shares common biological properties with other herpesviruses, such as persistence and latency in the host. Several lines of evidence indicate that both humoral and cellular immunity to CMV can reduce the frequency and severity of the disease [1–6]. So far, much importance has been placed on the role of cytotoxic T cells (CTL) in the recognition of CMV-infected cells, and the CD8^+^ CTL response to CMV antigens has been widely investigated using various reliable tools and methods [7–10]. There is however increasing evidence that CD4^+^ T cells also play a critical role in the control of CMV infection, reactivation and vertical transmission [3, 11]. However, the fine specificity pattern and nature of the CD4 response remain largely unsolved.

Due to its ability to elicit neutralizing antibodies (Abs) during infection, human CMV envelope gB is the leading subunit vaccine candidate [12–14]. A recent clinical report shows that the administration of a recombinant human CMV gB prevents maternal infection and tended to decrease congenital CMV infection [15]. Interestingly, a recent sub-study showed that both CMV-specific Abs and CD4^+^ T-cell responses can be boosted after vaccination with gB formulated in MF59, an oil-in-water emulsion, in women with chronic CMV infection [16]. Another study, attempting to understand the correlates of immune protection during the primary immune response to CMV, determined that the formation of effector memory CD4^+^ T cells was necessary for recovery from infection [17]. Thus, a deeper knowledge of the contribution of the CD4^+^ T-cell mediated immune response is essential to study the protective activity of the gB vaccine candidate. In the past few years, much research has focused on the use of tetramers, or on functional assays such as ELISPOT and intracellular cytokine staining (ICS) to explore CD4^+^ T cells responses in various disease settings or after vaccination in humans [18–21]. ELIPSOT and ICS assays commonly use overlapping pools of peptides to induce T cells to produce cytokines as an indication of their function. Although successful, these assays usually require several rounds of screening and so, depending on the size of the peptide library, might be costly, time-consuming and require large quantities of sample which may be unavailable.

By stimulating CD4^+^ T cells from CMV-seropositive donors with gB-primed autologous DCs and by using a simple and cost-efficient procedure based on the exogenous pathway to take up and process gB polypeptides expressed in recombinant bacteria, we derived several CD4^+^ T-cell clones and identified four gB epitopes presented by HLA-DR7, DP10 and DP2 molecules. The identification of gB-derived peptides such as those reported here may be of use in tracking clonal expansion of gB-specific CD4^+^ T cells during CMV infection and in monitoring the immune response of subjects vaccinated with recombinant gB or with recombinant vectors coding for gB, provided they allow for a significant coverage of the population on the basis of the frequency of the relevant HLA-class II alleles.

## Methods

### Media, Reagents and Cells

Culture media were RPMI (for EBV-B cells or dendritic cells (DCs)) and IMDM (for CD4^+^ T cells) supplemented with penicillin (100 U/ml), streptomycin (100 mg/ml), glutamine (2 mM) (Life Technologies, Gibco-BRL) and 10 % Fetal Bovine Serum (FBS) or 10 % human serum, respectively. The recombinant CMV gB used in the present work was obtained after mutagenesis to eliminate a cleavage site, and the transmembrane region was deleted to facilitate secretion of the glycosylated protein in Chinese hamster ovary cell culture [13]. Blood cells were collected at the Etablissement Français du Sang (EFS, Lyon, France) as buffy coat preparations from anonymous healthy, CMV-positive blood donors after informed consent and following EFS guidelines. Peripheral blood mononuclear cells (PBMCs) were isolated by density gradient centrifugation on Lymphoprep (Axix Shield PocAS, Oslo, Norway). Immature DCs were generated from CD14^+^ PBMCs isolated by positive selection using an anti-CD14 monoclonal antibody (mAb) coupled to magnetic microbeads (Miltenyi Biotech, Paris, France) and by sorting through a MACS® followed by 5 days of culture in complete RPMI medium supplemented with 10 U/ml IL-4 (Brucells, Brusssel, Belgium) and 50 ng/ml GM-CSF (PeproTech, Neuilly Sur Seine, France). Cultures were fed on days 2 and 4 by adding IL-4 and GM-CSF. CD4^+^ T lymphocytes were isolated from CD14^-^ PBMCs by positive selection using an anti-CD4 mAb coupled to magnetic microbeads (Miltenyi Biotech, Paris, France) and by sorting through a MACS®, as recommended by the manufacturer. The CD4^+^ T lymphocytes were kept frozen and were thawed the day before co-culture with DCs. Recombinant IL-2, IL-4, IL-6, IL-7 and IL-12 cytokines were purchased from AbCys S.A. (Paris, France). Anti-HLA DP, anti-HLA DR, anti-HLA DQ or anti-HLA (DP, DQ, DR) Abs and conjugated Abs anti-CD3-PE, antiCD4-PerCP-Cy5-5, anti-Granzyme B (GrB)-FITC were purchased from Becton Dickinson Biosciences (San Jose, California) and Antibodies-online GmbH (Aachen, Germany).

### Mixed Lymphocyte/Dendritic Cell Culture

Immature DCs (5 × 10^5^/ml) were incubated at 37 °C, 5 % CO_2_ for 18–20 h in complete medium supplemented with IL-4 (10 U/ml), GM-CSF (50 ng/ml) and a synthetic TLR4 agonist (1 ng/ml; EISAI, Japan) in the presence of recombinant gB (10 μg/ml). Cells were washed and added at 10^4^ per round-bottom microwell to 10^5^ autologous CD4^+^ T lymphocytes in 200 μl IMDM supplemented with 10 % human serum (hereafter referred to as complete IMDM) in the presence of IL-6 (1,000 U/ml) and IL-12 (10 ng/ml). The CD4^+^ T lymphocytes were stimulated on days 7, 14 and 21 with autologous DCs freshly loaded with gB and were grown in complete IMDM supplemented with IL-2 (10 U/ml) and IL-7 (5 ng/ml). The microcultures containing proliferating CD4^+^ T cells were then analyzed for their specificity at least 1 week after the last stimulation. Autologous EBV-B cells were incubated or not for 18–20 h in the presence of 10 μg/ml of recombinant gB, then washed and plated at 10^4^ cells per round-bottom microwell together with 3 × 10^3^ autologous CD4^+^ T lymphocytes in 200 μl of complete IMDM supplemented with IL-2 (25 U/ml). After 20 h, the supernatant was collected and the IFN-γ content was measured by ELISA (Becton Dickinson, San Jose, California). Inhibition with anti-DP, anti-DR, anti-DQ or anti-(DP, DQ, DR) Abs was performed by adding them at a concentration of 10 to 25 μg/ml during the experiment.

### CD4^+^ T-cell Clones

Cells from positive microcultures were cloned by limiting dilution, using as stimulator cells either mytomicin C -treated (Roche; 0.1 mg/ml PBS) or X-irradiated autologous EBV-B cells previously loaded with gB (5 × 10^3^ to 2 × 10^4^ cells). CD4^+^ T-cell clones were grown in complete IMDM supplemented with IL-2 (50–150 U/ml) and occasionally IL-7 (5 ng/ml). The clones were divided and supplemented with fresh culture medium and stimulator cells (2 × 10^5^ per well in a 48-well plate) at 1- to 2-week intervals. Established CD4^+^ T-cell clones were tested for IFN-γ production after stimulation with autologous EBV-B cells pulsed with either gB, recombinant bacteria expressing the full gB or its sub-fragments, or gB-derived synthetic peptides.

### Construction of pGEX and pET28c Containing gB-cDNA

The full sequence encoding recombinant human CMV gB [13] was cloned into the pET28c expression vector (Novagen, EMD Chemicals, Inc. Gibbstown, US). Then, four overlapping fragments of the gB coding sequence (gB_1-218_, gB_199-417_, gB_398-617_ and gB_598-806_ hereafter referred as to A, B, C and D, respectively) were subcloned into the pGEX-4T-1 plasmid using gB-specific primers containing the *NotI* and *BamHI* restriction sites in fusion with the glutathione serine transferase (GST) [22]. Each of the four sequences, A, B, C and D, was then subcloned by PCR into pGEX-4T-1 in five overlapping sequences coding for peptides of ~60 amino acids (aa) that overlap by ~20 aa (hereafter referred as to A1 to A5, B1 to B5, C1 to C5 and D1 to D5, respectively). The PCR products were purified using a QIAquick PCR purification kit (Qiagen, Courtaboeuf, France), digested using the *BamHI* and *NotI* restriction enzymes and the inserts were cloned into the pET28c plasmid using the Rapid DNA ligation kit (Roche). Plasmids were purified with the Biorobot 9600 using the NucleoSpin® Plasmid extraction kit (Qiagen), and the sequence of the inserts was systematically checked. *E. coli* BL21 bacteria were transformed by electroporation with the various recombinant plasmids and selected with ampicillin (50 μg/ml).

### Recombinant Bacteria Recognition Assay

The procedure used to identify the natural peptide recognized by each of the CD4^+^ clones was based on the exogenous pathway to take up and process polypeptides encoded by overlapping gB cDNA fragments expressed in recombinant BL21 bacteria. To obtain a standard amount of bacteria containing the various fragments of gB cDNA, the recombinant bacteria representing each gB polypeptide were amplified in LB medium containing ampicillin (50 μg/ml) and kanamycin (7.5 μg/ml), at 37 °C under agitation until an OD_600_ of 0.5 was reached. Isopropyl β-D-thiogalactoside (IPTG; 1 mM) was added to induce protein expression. Incubation was continued under agitation for 4 h at 37 °C, and the recombinant bacteria were then centrifuged at 2500 rpm for 15 min. The supernatant was discarded and the bacteria were suspended in PBS and kept frozen at −80 °C until use. The recombinant bacteria (5 μl) expressing each gB sub-fragment were added to EBV-B cells and plated out in 96-well U-bottom plates (2 × 10^4^ cells/well) in 50 μl IMDM/10 % FBS supplemented with 20 μg/ml gentamycin. The CD4^+^ T-cell clones (3 to 5 × 10^3^ cells/well) were added extemporaneously in 100 μl IMDM/10 % human serum supplemented with IL-2 (25 U/ml) and gentamycin (20 μg/ml). After 18 h of co-culture, the supernatants were harvested and the amount of IFN-γ released by the specific CD4^+^ T-cell clones was measured using a standard ELISA.

### Recognition Assays with Peptides or with Towne Virus Infected Cells

Peptides were synthesized on solid phase using F-moc for transient NH_2_-terminal protection and were characterized using mass spectrometry (Neosystem, Strasbourg, France). All peptides were controlled by mass spectrometry and were ≥80 % pure, as indicated by analytical HPLC. Lyophilized peptides were kept frozen after dissolution in DMSO. CD4^+^ T-cell clones were incubated with either autologous HLA-matched or HLA-mismatched EBV-B cells that had been pre-incubated for 2 h with each individual peptide, the indicated concentrations representing their concentrations during the incubation step. They were distributed at 20 000 cells per round-bottom microwell together with 3 to 5 × 10^3^ CD4^+^ T lymphocytes in 150 μl of complete IMDM supplemented with IL-2 (20 U/ml). Occasionally, CD4^+^ T-cell clones were incubated with autologous EBV-B cells infected with the Towne CMV laboratory strain at a multiplicity of infection of 10:1 for 1 h, 24 h prior to use. The supernatants were harvested after 18 to 36 h and assayed for IFN-γ by ELISA.

### Flow Cytometry

For analysis of expression of surface markers, a total of 2 × 10^5^ cells were incubated with fluorescent labeled conjugated mAbs according to manufacturer’s instructions for 30 min at 4 °C. Samples were fixed and permeabilized (BD Biosciences) and then stained for GrB expression according to the recommended procedure. Flow cytometry analysis was performed by FACSCalibur flow cytometer and CellQuest software (BD Biosciences).

### HLA-class II Genotyping

HLA-class II genotyping was performed by PCR from genomic DNA according to Dynal Biotech procedures (Dynal AllSet+™ SSP DR “low resolution”) by C.Ris Pharma (Saint Malo, France) [23].

## Results

**Fig. 1:**
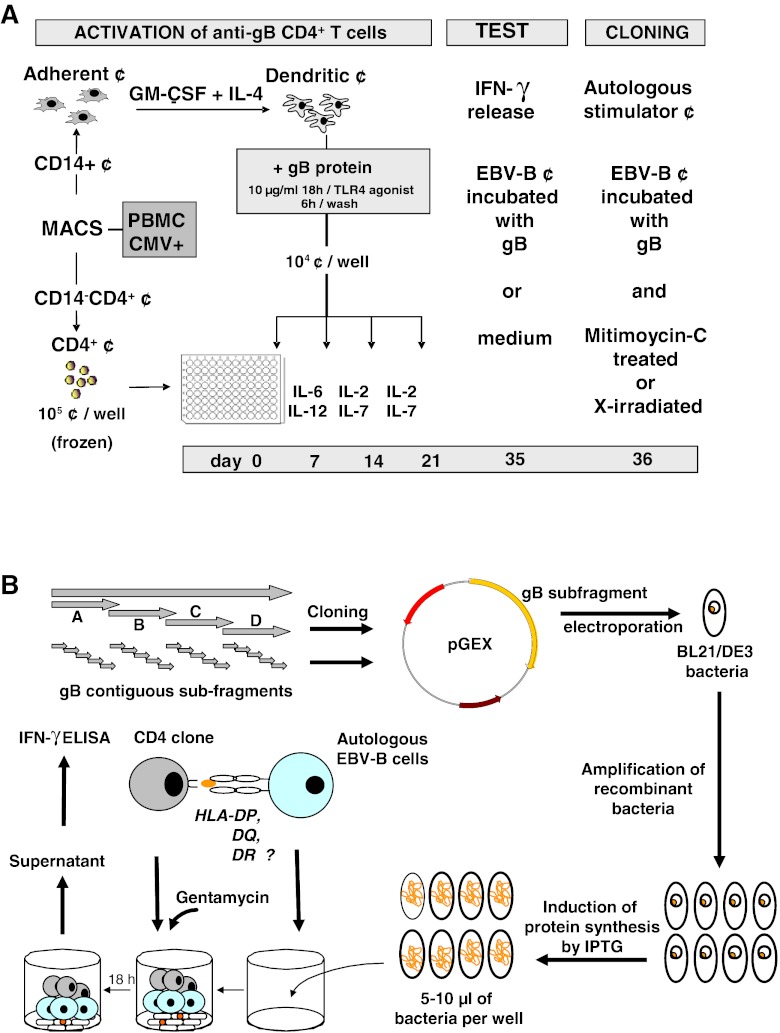
Overview of the procedure used to obtain and characterize anti-gB CD4^+^ T-cell clones

The experiments were performed with blood cells from two CMV-seropositive donors, using CD4^+^ T lymphocytes as responder cells and autologous DCs loaded with recombinant gB as stimulator cells. Responder CD4^+^ T cells were stimulated at weekly intervals. After a resting period of 2 weeks, the microcultures that specifically produced high levels of IFN-γ after stimulation with autologous EBV-B cells loaded with gB were cloned by limiting dilution in the presence of IL-2. EBV-B cells loaded with gB were either irradiated or mitomycin-C treated and used as stimulating cells for the cloning step because of the limited supply of autologous DCs. After a few weekly re-stimulations, growing CD4^+^ clones were tested for their ability to recognize autologous EBV-B cells loaded with gB and were further characterized (Fig. 1a). In order to identify the natural gB-peptide recognized by CD4^+^ T cell clones, recombinant *E. coli* expressing full gB or overlapping gB-polypeptides were produced. As a first step, human CMV-gB cDNA fragments was cloned into the pGEX expression vector and used to transform *E. coli.* After induction of protein synthesis, recombinant bacteria were incubated directly with autologous EBV-transformed B cells that were subsequently used to stimulate autologous CD4^+^ clones. The use of minigenes coding for gB fragments expressed in recombinant bacteria precisely pinpointed the region encoding the antigenic peptide recognized by a given CD4^+^ clone, and avoided the synthesis and the screening of a large library of overlapping peptides (Fig. 1b).

### Clone 1A10 Recognizes Peptide PDDYSNTHSTRYVTV on HLA-DR7

**Fig. 2:**
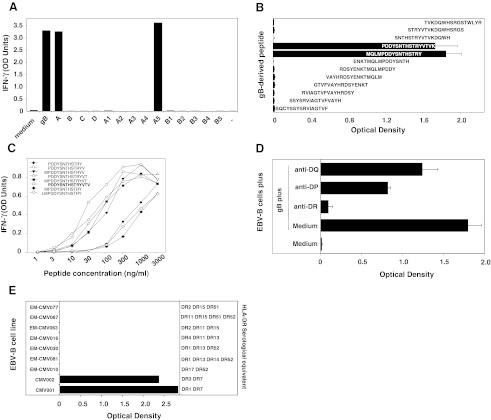
Characteristics of clone 1A10. **a** Recognition of EBV-B cells loaded with recombinant gB-expressing bacteria by CD4^+^ T-cell clone 1A10. CD4^+^ T-cell clone 1A10 (3000 cells) were co-cultured for 20 h with autologous CMV001 EBV-B cells (20 000 cells/well) loaded or not with gB (10 μg/ml) or with recombinant bacteria that expressed each of the 4 overlapping gB constructs (A, B, C, D) or each of five overlapping constructs spanning the A (A1 to A5), B (B1 to B5), C (C1 to C5) or D (D1 to D5) fragments. The amount of IFN-γ secreted in the supernatant of the co-cultures was measured using standard ELISA. The results shown represent an average of duplicate co-cultures. **b** Recognition of gB overlapping peptides by CD4^+^ T-cell clone 1A10. EBV-B cells were incubated for 2 h with each of the gB overlapping peptides (1 μg/ml). CD4^+^ T-cell clone 1A10 (3000 cells) were then co-cultured with autologous peptide-pulsed EBV-B cells (20 000 cells) for 18 h. IFN-γ production in the supernatant was measured by ELISA. The results shown represent the average of triplicate cultures. **c** Identification of the minimal gB epitope recognized by CD4^+^ T-cell clone 1A10. EBV-B cells were distributed in microwells (2 × 10^4^ cells) and incubated for 2 h with various concentrations of each individual peptide. A total of 3 to 5 × 10^3^ cells from autologous CD4^+^ T-cell clone 1A10 was added and the presence of IFN-γ production in the supernatant was measured by ELISA after overnight culture. The results shown represent an average of duplicate cocultures. **d** Inhibition of the gB peptide recognition by anti-HLA-DP, -DQ or -DR Abs. Autologous EBV-B cells were incubated with 10 μg/ml of recombinant gB for 20 h. CD4 T-cell clones (3 000 cells) were then co-cultured with 20 000 protein-loaded EBV-B cells for 20 h. Inhibition with anti-HLA class II mAbs was performed by addition of 10 μg/ml of mAb during the co-culture. IFN-γ production was measured by ELISA after 18 h. **e** HLA-class II restriction analysis of the gB epitope. The gB-specific CD4^+^ T-cell clone 1A10 was exposed to gB-loaded autologous or allogeneic EBV-B cells. IFN-γ production was measured by ELISA after overnight co-culture. The results shown represent the average of triplicate co-cultures

Clone 1A10 was obtained by limiting dilution from a microculture of CD4^+^ T lymphocytes from donor CMV001, using autologous EBV-B cells loaded with gB as stimulator cells. Clone 1A10 recognized autologous EBV-B cells loaded either with gB or with recombinant *E. coli* bacteria expressing either fragment A (gB_1-218_) or sub-fragment A5 (gB_158-218_) (Fig. 2a). To precisely identify the gB peptide recognized by the clone, we screened a set of 12 synthetic peptides of 16 aa, overlapping by 12 aa and covering the gB_158-218_ sequence. Autologous EBV-B cells were incubated with each individual peptide at a concentration of ~1 μM and were tested for their recognition by CD4^+^ T-cell clone 1A10 in an IFN-γ ELISA. Clone 1A10 recognized two overlapping peptides, namely MQLMPDDYSNTHSTRY (gB_186-201_) and PDDYSNTHSTRYVTVK (gB_190-205_) (Fig. 2b). Unlike peptides presented by HLA-class I molecules, those presented by HLA-class II usually vary in length and tolerate extensions at both the NH_2_ and COOH termini because they are not attached by their ends in the HLA groove. It is therefore difficult to define the length of the peptides precisely. We tested a large number of gB peptides of different lengths and concluded that the optimal sequence of the peptide recognized by clone 1A10 is PDDYSNTHSTRYVTV (gB_190-204_). Half-maximum value of IFN-γ production was obtained by incubating stimulator cells with ~30 ng/ml of this peptide (~17 nM) (Fig. 2c). This compares favorably with the results obtained with other epitopes recognized on HLA-class II molecules by CD4^+^ T cells, and this is in accordance with the IC_50_ prediction value of 33 nM determined using SMM-align, a stabilization matrix alignment method available online (http://tool.immuneepitope.org). Recognition by clone 1A10 of cells loaded with gB was abolished by an anti-DR Ab (Fig. 2d). The serological equivalent for HLA genotyping of donor CMV001 was DR1 DR7 DR53. To identify the HLA-DR molecule that presents the gB-derived peptide to clone 1A10, the gB_190-204_ peptide was loaded on a panel of partially HLA-matched or mismatched EBV-B cell lines. All and only peptide-sensitized cell lines expressing HLA-DR7 were recognized by clone 1A10 (Fig. 2e). Of note, this immunogenic peptide was confirmed as being naturally processed and presented by HLA-DR7 utilizing the ProPresent™ technology, a mass spectrometry-based analysis (data not shown) (ProImmune, Oxford, UK). The PDDYSNTHSTRYVTV sequence contains a gB epitope that has in fact been previously identified by other investigators. Elkington et al. isolated a human cytotoxic CD4^+^ T-cell clone that recognized the DYSNTHSTRYV peptide (gB_192-202_) in association with HLA-DR7 [24]. This 11-aa sequence, named DYS, was the most active in a CTL assay under limiting dilution, and DYS-specific CD4^+^ T cells showed extreme conservation of T-cell receptor usage, which suggests a strong clonal selection for its recognition [25]. HLA-DR7 is expressed in ~23 % of Caucasians, ~25 % of Africans and ~6 % of Asians.

### Clone 2G12 Recognizes Peptide HSRSGSVSQRVTSSQ on HLA-DP10

**Fig. 3:**
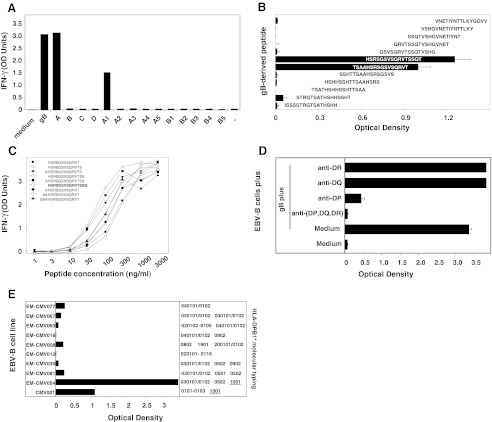
Characteristics of clone 2G12. The procedure used for clone 2G12 was the same as the one described in Fig. 2. **a** Recognition of EBV-B cells loaded with recombinant gB-expressing bacteria by CD4^+^ T-cell clone 2G12. **b** Recognition of gB overlapping peptides by CD4^+^ T-cell clone 2G12. **c** Identification of the minimal gB epitopes recognized by CD4^+^ T-cell clone 2G12. **d** Inhibition of the gB peptide recognition by anti-HLA-DP, -DQ or -DR Abs. **e** HLA-class II restriction analysis of the gB epitope recognized by CD4^+^ T-cell clone 2G12

A second CD4^+^ T-cell clone with a different specificity was obtained from the microcultures of CD4^+^ T lymphocytes from donor CMV001. Clone 2G12 recognized autologous EBV-B cells loaded with gB, or with recombinant bacteria expressing either gB fragment A (gB_1-218_) or sub-fragment A1 (gB_1-57_) (Fig. 3a). To identify the gB peptide recognized by clone 2G12, a set of 12 peptides of 16 aa was screened. The peptides overlapped by 12 aa and covered the gB_1-57_ sequence. CD4^+^ T-cell clone 2G12 recognized two overlapping peptides, namely TSAAHSRSGSVSQRVT (gB_18-33_) and HSRSGSVSQRVTSSQT (gB_22-37_) (Fig. 3b). In order to define the minimal peptide recognized by clone 2G12, a set of 9 overlapping peptides from 12 to 15 aa was screened. The nine overlapping peptides were all recognized by clone 2G12 and stimulated strong production of IFN-γ, although peptide HSRSGSVSQRVTSSQ (gB_22-36_) showed the strongest activity and was the most active in limiting dilution (Fig. 3c). We concluded that the shortest peptide well recognized by clone 2G12 is gB_22-36_. Half-maximum value of IFN-γ production was obtained by incubating stimulator cells with ~50 ng/ml of this peptide (~30 nM). Recognition by clone 2G12 of cells loaded with gB was abolished by an anti-HLA-(DP, DQ, DR) and also by an anti-DP Ab, but not by anti-DR or DQ specific Abs (Fig. 3d). CMV001 donor was typed DP1 DP10. To identify the HLA-DP molecule that presents the gB peptide recognized by clone 2G12, gB was loaded on a panel of allogeneic EBV-B cell lines. All and only the peptide-sensitized HLA-DP10 cell lines tested were recognized by clone 2G12 (Fig. 3e). HLA-DP10 is expressed in ~3 % of Caucasians and ~1 % of Asians.

### Clone 2C4 Recognizes FETTGGLVVFWQGIK, a Peptide with a Strong Affinity for HLA-DR7

**Fig. 4:**
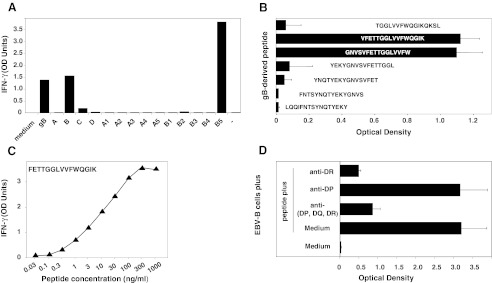
Characteristics of clone 2C4. The procedure used for clone 2C4 was the same as the one described in Figs. 2 and 3. **a** Recognition of EBV-B cells loaded with recombinant gB-expressing bacteria by CD4^+^ T-cell clone 2C4. **b** Recognition of gB overlapping peptides by CD4^+^ T-cell clone 2C4. **c** Identification of the minimal gB epitope recognized by by CD4^+^ T-cell clone 2C4. **d** Inhibition of the gB peptide recognition by anti-HLA-DP, -DQ or -DR Abs

A third CD4^+^ T-cell clone, called 2C4, was obtained from CMV001 donor, and was also very well stimulated by cells loaded with gB or with recombinant bacteria expressing either B (gB_199-417_) or B5 polypeptides (gB_359-417_) (Fig. 4a). We screened a set of 7 synthetic overlapping peptides and showed that clone 2C4 recognized GNVSVFETTGGLVVFW (gB_391-406_) and VFETTGGLVVFWQGIK (gB_395-410_) (Fig. 4b). The binding prediction (http://tool.immuneepitope.org) analysis indicated that the gB_396-410_ peptide (FETTGGLVVFWQGIK) was a very good binder for HLA-DRB1*0701 (score 231659.5 versus 307.2 for HLA-DRB1*0101). Half-maximum value of IFN-γ production was obtained by incubating the stimulator cells with 10 ng/ml of this peptide (~6 nM) (Fig. 4c). In accordance with these data, recognition by clone 2G12 of cells loaded with the gB_396-410_ peptide was abolished by an anti-HLA-(DP, DQ, DR) and also by an anti-DR Ab, but not by an anti-DP Ab (Fig. 4d), suggesting that gB_396-410_ is presented to clone 2C4 in association with HLA-DR7. Unfortunately, we were unable to expand this clone *in vitro* to further confirm the HLA-class II presenting molecule.

### Clone B3A5 Recognizes Peptide YEYVDYLFKRMID on HLA-DP2

**Fig. 5:**
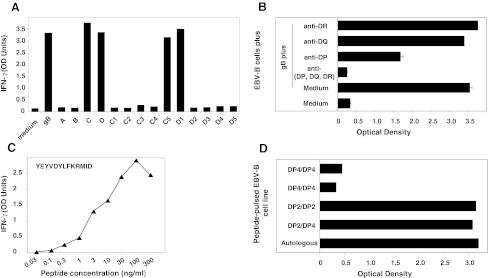
Characteristics of clone B3A5. The procedure used for clone B3A5 was the same as the one described in Figs. 2 and 3. **a** Recognition of EBV-B cells loaded with recombinant gB-expressing bacteria by CD4^+^ T-cell clone B3A5. **b** Inhibition of the gB peptide recognition by anti-HLA-DP, -DQ or -DR Abs. **c** Identification of the minimal gB epitopes recognized by by CD4^+^ T-cell clone B3A5. **d** HLA-class II restriction analysis of the gB epitope recognized by CD4^+^ T-cell clone B3A5

Clone B3A5 was obtained from CMV016, a second CMV-seropositive donor. It recognized autologous EBV-B cells loaded with gB or with recombinant bacteria expressing C (gB_398-617_) and D (gB_598-806_) gB-overlapping fragments. Accordingly, the 2 overlapping sub-fragments C5 (gB_558-617_) and D1 (gB_598-657_) also induced high levels of IFN-γ secretion (Fig. 5a). The C5 and D1 polypeptides shared the 21-aa peptide sequence NSAYEYVDYLFKRMIDLSSI (gB_598-617_) that therefore contained the optimal sequence of the natural peptide recognized by clone B3A5. The recognition of gB-loaded cells by CD4^+^ clone B3A5 was inhibited by an anti-HLA-(DP, DQ, DR) and by anti-HLA-DP Abs, but not by anti-DR or anti-DQ specific Abs (Fig. 5b). CMV016 donor was typed HLA-DP2 DP4. Interestingly, HLA-DP4 and HLA-DP2 were suggested to form a supertype of HLA-class II molecules on the basis of homology in the peptide-binding pockets P1 and P6 and are expected to share a common repertoire of peptides [26]. The HLA-DP4 peptide-binding site consists of two large aromatic pockets at positions 1 (P1) and 6 (P6) that accommodate aromatic/hydrophobic residues such as Phe, Tyr, and Leu. Based on this model, the 13-aa peptide, YEYVDYLFKRMID (gB_601-613_), is thought to contain three putative HLA-DP4 binding motifs, the major P1, P4 and P6 anchors being Tyr, Tyr and Phe, respectively [26]. Therefore, gB_601-613_ appeared as the minimal gB-derived epitope recognized by clone B3A5 in association with HLA-DP. Peptide titration analysis revealed a high avidity with a half-maximum value of IFN-γ production obtained by incubating the stimulator cells with ~10 ng/ml of this peptide (~6 nM) (Fig. 5c). These observations suggested that the gB_601-613_ peptide could be presented in association with HLA-DP4 and/or HLA-DP2 to anti-gB CD4^+^ T cell clones. Attempts to label either PBMCs isolated from donor CMV016 as well as CD4^+^ T-cell clone B3A5 using a PE-conjugated HLA-DP4 tetramer loaded with the YEYVDYLFKRMID peptide (kindly provided by Dr. E. James, the Benaroya Research Institute, Seattle, [27]) failed (data not shown). These data suggest that, for donor CMV016, the YEYVDYLFKRMID epitope is strictly presented in association with HLA-DP2. In line with these data, all gB-loaded HLA-DP2 cell lines were recognized by B3A5 whereas homozygous HLA-DP4 EBV-B cells pulsed with the YEYVDYLFKRMID peptide did not stimulate B3A5 (Fig. 5d). HLA-DP2 is expressed in ~25 % Caucasians whereas HLA-DP4 is expressed in ~60 %. Whether this peptide is presented in association with HLA-DP4 molecules in other CMV-seropositive subjects deserves further investigation.

### Presentation of CMV gB to CD4^+^ T Cells by EBV-B Cells Infected with the Towne CMV Strain

**Fig. 6:**
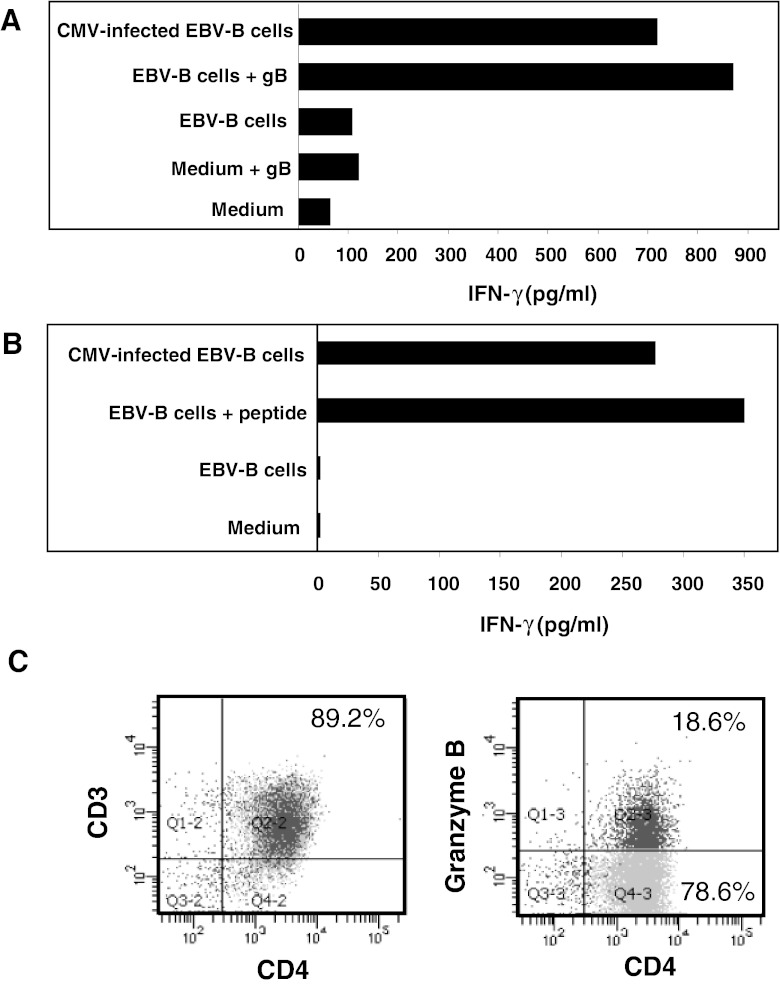
Recognition of HCMV-infected cells by two CD4^+^ T-cell clones directed against gB and expression of GrB by clone B3A5. gB-specific CD4^+^ T-cell clones B3A5 (**a**) or 1A10 (**b**) recognize autologous EBV-B cells infected with the Towne laboratory strain. CD4^+^ T-cell clones were stimulated with autologous EBV-B cells, loaded or not either with a recombinant gB protein or with the relevant gB-derived peptide, or infected with the Towne CMV strain (10 pfu/cell). IFN-γ production in the supernatant was measured by ELISA after 36 h. (**c**) Anti-GrB labeling of clone B3A5. Percentages of CD3^+^CD4^+^ or CD4^+^GrB^+^ double positive cells are indicated in the upper right corner of each subpanel

One essential feature of functional CD4^+^ T cells is their ability to recognize naturally processed antigens. EBV-B cells, known to express HLA-class II molecules, infected with the Towne CMV laboratory strain stimulated the gB-specific clones B3A5 and 1A10 to produce IFN-γ at levels similar to that induced by autologous EBV-B cells that present either soluble gB or the relevant gB-derived peptide (Fig. 6a and b). Our results are in accordance with published data showing that gB synthesized endogenously in CMV-infected cells can reach the HLA-class II presentation pathway [28] and suggest that the gB-derived epitopes sorted to endosomes of HLA-class II of CMV-infected cells are efficiently presented to anti-gB CD4^+^ T-cell clones *in vivo*. Interestingly, we showed that around 20 % of CD4^+^ T-cell clone B3A5 expressed the cytolytic molecule GrB, suggesting that clone B3A5 is capable of performing cytolysis through this pathway (Fig. 6c).

## Discussion

A comprehensive understanding of the human CD4^+^ T-cell response to CMV remained hampered by limitations in the techniques and tools used in assaying for specific CD4^+^ T cells. The CMV-specific response in immunocompetent subjects has been evaluated by ICS following stimulation of CD4^+^ T cells with crude extracts of CMV proteins prepared from infected cells or with peptide libraries and has revealed broad antigen recognition [29, 30]. When overlapping 15-mer peptides comprising all known CMV open reading frames were used to stimulate CD4^+^ T cells in CMV-infected subjects, 125 of 213 (58.7 %) of the open reading frames were found to be recognized by either CD4^+^ only or by both CD4^+^ and or CD8^+^ T cells [30]. In healthy CMV-seropositive individuals, the frequency of CMV-specific CD4^+^ memory T cells has been reported to reach a median of 9.1 % [30]. This frequency reached even 40 % in some donors suggesting that, as for CD8^+^ CTL, high frequencies of CMV-specific CD4^+^ T cells are mandatory to control CMV reactivation [31]. Accordingly, gB-specific cytotoxic CD4^+^ T cells have been successfully isolated and expanded *in vitro* [24, 28].

In clinical trials involving a recombinant gB, it will be essential to have reliable monitoring of the anti-gB CD4^+^ response. Several strategies to identify HLA-class II epitopes can be considered. A first classical approach is based on the location of candidate peptides carrying consensus anchor motives for a certain HLA in the protein sequence by *in silico* algorithms [32]. However, the selection of these candidate peptides harboring anchor residues is limited by less accurate predictive methods of HLA-class II binding peptides as compared to HLA-class I binding peptides. Moreover, the accuracy of these predictions must be confirmed empirically [32], as peptide binding to the HLA molecules does not always guarantee that the epitope is naturally processed. For example, CD4^+^ T cells specific for MAGE-A3_161-175_ or for MAGE-A3_171-185_ peptides were obtained upon *in vitro* stimulation of PBMCs from melanoma patients. Recognition of the respective native epitopes was tested by recognition of autologous EBV-B cells engineered to express MAGE-A3 either in the cytoplasm or in the endosomal/lysosomal compartment and the HLA-class II positive autologous melanoma. These CD4^+^ T cells failed to recognize the naturally processed epitope, suggesting that the MAGE-A3_161-175_ and MAGE-A3_171-185_ peptides do not contain natural epitopes produced after processing through both the endogenous and the exogenous pathways [33]. We produced gB-polypeptides using a simple, cost saving bacterial expression system and used the exogenous processing pathway of autologous EBV-B cells to identify the naturally processed gB-peptide recognized by established CD4^+^ T cell clones. This technique offers the advantage of avoiding the synthesis of a peptide library, for which the size and cost can be prohibitive, and is mainly applicable for the determination of CD4^+^ T-cell epitopes derived from large proteins or from proteins of unknown sequence derived from a cDNA expression library [34]. Morevoer, the isolated CD4^+^ T-cell clones described here have additional interest, as their T-cell receptors (TCRs) might be used both for diagnostic and therapeutic purposes. Firstly, the soluble TCRs might help identifying and tracing HLA-peptide complexes on the surface of CMV-infected cells and tissues. Furthermore, gB-specific TCR-transgenic CD4^+^ T cells could have clinical application. The development of a CMV-specific T-cell therapy in hematopoietic stem cell (HSC) transplant is indeed highly warranted, because patients transplanted with allogeneic HSC from CMV-seronegative donors are at high risk of developing CMV-related disease. Transgenic pp65-specific CD8^+^ T cells were already successfully generated from primary T cells of CMV-seronegative donors by retroviral TCR transfer [35]. In concert with pp65 specific-TCR transgenic CD8^+^ T cells, gB-specific TCR-transgenic T cells could represent promising tools for adoptive T cell therapy in HSC transplant patients.

To our knowledge, the characterization of established gB-specific CD4^+^ clones has led to the identification of a single gB-derived epitope, namely DYSNTHSTRYV (DYS), which is restricted by HLA DRB*0701 [24]. In the present study, we have isolated several human CD4 clones that recognize epitopes from CMV-gB restricted by HLA-DR7, HLA-DP1 and HLA-DP2 molecules. The gB_190-204_ epitope identified in this study contains the DYS epitope that has already been reported to be presented by HLA-DR7 [24]. Surprisingly, the gB_601-613_ peptide, which contains the four known anchor residues common for HLA-DP2 and HLA-DP4 [26], was only presented by HLA-DP2 to the relevant CD4 clone. Given the high allele frequencies of HLA-DPB1*0401 and DPB1*0402 worldwide, the binding of this epitope and its recognition by anti-gB CD4^+^ T cells in HLA-DP4 subjects deserves further investigation, as this would open the possibility of using this peptide for the purposes of clinical monitoring of a large proportion of subjects vaccinated with gB. Obviously, further identification of gB-derived epitopes to cover additional HLA allotypes is highly desirable.

Remarkably, two of the anti-gB CD4^+^ T-cell clones established in this work recognized autologous EBV-B cells infected with the Towne laboratory strain, suggesting that the gB epitopes have access to the endogenous HLA-class II presentation pathway of the CMV-infected cells and that the recognition process of HLA-class II infected cells could occur *in vivo*. The exact mechanism governing the presentation of endogenous gB products on HLA-class II molecules still remains to be elucidated. Our data are in accordance with recent data showing that gB can be presented directly from CMV-infected HLA-class II cells to gB-specific CD4^+^ cytotoxic T cells, able to kill target cells without the need for cross-presentation [24, 28]. Our results illustrate the possibility to follow the amplification of such clones of cytotoxic CD4^+^ T cells, which should be considered as important in the design of vaccine strategies.

## Conclusion

The generation of appropriate CD4^+^ T-cell responses that are likely to help increase baseline gB-specific antibody levels should be considered and would require additional evaluation in future clinical trials. To that end, we have applied a simple and broadly applicable method to characterize several novel, naturally processed CD4^+^ T-cell epitopes from the CMV gB protein that will help investigating the role of natural and vaccine-induced CD4^+^ T-cell responses against CMV. Finally, our data may contribute to analysis of the threshold of the anti-gB CD4^+^ T-cell immune response required in protection against congenital CMV infection.
